# Evaluating the Potential of Q-Band ESR Spectroscopy for Dose Reconstruction of Fossil Tooth Enamel

**DOI:** 10.1371/journal.pone.0150346

**Published:** 2016-03-01

**Authors:** Verónica Guilarte, François Trompier, Mathieu Duval

**Affiliations:** 1 Centro Nacional de Investigación sobre la Evolución Humana (CENIEH), Burgos, Spain; 2 Institut de Radioprotection et de Sûreté Nucléaire (IRSN), Fontenay-aux-Roses, France; Université de Poitiers, FRANCE

## Abstract

The potential of Q-band Electron Spin Resonance (ESR) for quantitative measurements has been scarcely evaluated in the literature and its application for dose reconstruction of fossil tooth enamel with dating purposes remains still quite unknown. Hence, we have performed a comparative study based on several Early to Middle Pleistocene fossil tooth samples using both X- and Q-band spectroscopies. Our results show that Q-band offers a significant improvement in terms of sensitivity and signal resolution: it allows not only to work with reduced amounts of valuable samples (< 4 mg), but also to identify different components of the main composite ESR signal. However, inherent precision of the ESR intensity measurements at Q-band is clearly lower than that achieved at X-band, highlighting the necessity to carry out repeated measurements. All dose values derived from X- and Q-band are nevertheless systematically consistent at either 1 or 2 sigma. In summary, our results indicate that Q-band could now be considered as a reliable tool for ESR dosimetry/dating of fossil teeth although further work is required to improve the repeatability of the measurements.

## Introduction

The use of Electron Spin Resonance (ESR) spectroscopy (also known as Electron Paramagnetic Resonance: EPR) for dating purposes was first suggested by Ikeya [1], who published a pioneering work based on the study of a few stalactites from Japanese caves. Since then, numerous dating applications to a wide range of materials have been investigated (see [2–4] for extensive reviews). Among them, fossil tooth enamel is one of the most interesting materials because of its excellent dosimetric properties to register doses from around 100 mGy to several thousands of Gy [5,6], together with the high stability of the radiation-induced centers (~ 10^9^ years at 25°C) [7].

ESR dose reconstruction of fossil tooth enamel for dating purpose is traditionally performed with X-band spectrometers (υ ~ 9–10 GHz), mainly because they offer a good compromise between sensitivity and measurement repeatability and are available at most universities and research centers. The standard procedure in ESR dating of tooth enamel is based on the Multiple Aliquots Additive (MAA) dose approach, which consists in dividing the natural sample into different aliquots that are irradiated at increasing doses (e.g. [5]). For practical reasons, ESR measurements are almost exclusively performed on fossil enamel powder, using usually at least several tens of milligrams per aliquot. Consequently, a total of several hundreds of milligrams of enamel sample may be required for the dating process, which is problematic when working on tooth samples with a small amount of enamel available, e.g. that are either fragmented, with a thin enamel layer, or highly valuable fossil hominin remains.

In the last years, the progress of microwave technology has facilitated the use of other resonant frequencies. Among them, Q-band ESR spectrometers (υ ~ 34 GHz) are becoming increasingly popular because of their higher spectral resolution [8], the magnitude of the magnetic field being about four times higher at Q-band than at X-band. Consequently, Q-band ESR spectrometers can resolve peaks with very close g-values that could not be separated at X-band, which may be especially useful for the study of strongly composite signals, such as those of fossil tooth enamel [9–11]. In addition, the sensitivity of an ESR spectrometer increases as a function of ~ υ^2^: Q-band resonators thus require a much smaller sample mass (i.e., only a few milligrams, < 5 mg, [12,13]) in comparison with X-band equipments.

However, Q-band resonators have a smaller size, resulting in a much smaller effective sample volume in comparison with their X-band equivalent. As the signal intensity is proportional to mass, X-band remains preferred for large sample mass: detection limit for enamel being of about 100 mGy at X-band using 100 mg against 400 mGy at Q-band with 5 mg sample mass [14]. In addition, there are also considerable practical difficulties in using Q-band spectroscopy for dosimetric/dating purpose, such as a lower reproducibility of sample positioning in the cavity, as well as a higher sensitivity of the resonator’s response to the experimental conditions [15]. Due to these drawbacks, the number of ESR dosimetry/dating applications involving Q-band spectroscopy has actually been quite limited over the last decade. In the field of retrospective dosimetry, the first paper related to Q-band ESR was published by Romanyukha in 2007 [12] and only a few studies have been conducted until now, mostly involving modern tooth enamel [13,14] and fingernails [16–18]. Regarding fossil tooth enamel, most of the published works are based on qualitative studies of the nature and composition of the ESR signals associated to hydroxyapatite [11,15,19–21]. So far, the very few attempts of quantitative analysis described in the literature have not produced really encouraging results: they have showed measurements with low precision and reproducibility, inducing dose estimations with a considerably large experimental error (~ 30%) [10,22]. Consequently, the real potential of Q-band ESR spectroscopy for dating tooth enamel remains still to be fully evaluated. To address this issue, we performed a first comparative study based on several fossil tooth samples from various Pleistocene archaeological and/or paleontological sites. Dose response curves (DRCs) were measured with both Q-band and X-band ESR spectrometers at the Institut de Radioprotection et de Sûreté Nucléaire (IRSN, Fontenay-aux-Roses, France) and at the Centro Nacional de Investigación sobre la Evolución Humana (CENIEH, Burgos, Spain), respectively.

## Materials and Methods

No permits were required for the described study, which complied with all relevant regulations.

We selected three fossil tooth samples coming from different Early to Middle Pleistocene sites and covering a wide range of equivalent dose (D_E_) values (between > 100 and < 2000 Gy): sample #1 was collected at the late Middle Pleistocene locality of Tourville-la-Rivière (France) [23], while sample #2 is from the Middle Pleistocene site of Cuesta de la Bajada (Spain) [24] and fossil tooth #3 was sampled at the Early Pleistocene site of Venta Micena (Spain) [25].

Fossil teeth were prepared following a standard ESR dating protocol described in [25]: the three different tissues (cementum, enamel and dentin) were separated mechanically. The enamel layer was cleaned using a dentist drill, and then ground and sieved to recover the size fraction 100–200 μm. Enamel samples were analysed following a MAA dose method. Depending on the available mass of enamel powder, each sample was split into 10–14 aliquots. These aliquots were irradiated at increasing doses with a calibrated ^60^Co gamma source, using an exponential dose step distribution [26].

X-band ESR measurements were carried out using a EMX micro 6/1 Bruker spectrometer coupled to a standard rectangular TE_102_ cavity (ER 4102ST). To ensure constant experimental conditions over time, the temperature of the water circulating in the magnet was controlled and stabilized at 18°C by a water-cooled Thermo Scientific NESLAB ThermoFlex 3500 chiller, and the temperature of the room was kept constant at 20°C. ESR measurements were performed at room temperature using the acquisition parameters shown in **Table 1**. In order to ensure similar resonance conditions in the resonator for all the aliquots of a given sample (i.e. the natural aliquot plus the gamma irradiated ones), each aliquot was carefully weighed in a tube. Depending on the enamel sample, the mass per aliquot was ranging from 68 to 90 mg. A maximum variation of 1 mg was tolerated for all the aliquots from a given sample (i.e. corresponding to a relative variability of 1% in weight). A special attention was paid to the optimisation of the vertical position of the sample in the cavity, so that the centre of the aliquot matches the centre of the cavity [5,27]. Basically, all aliquots of a given sample were successively measured in a short time (~ 1 h), ensuring thus stable experimental conditions. This procedure was repeated over several days without removing the enamel from the ESR tubes between measurements. Intensities were extracted from peak-to-peak amplitude measurements of the ESR signal of enamel (T1-B2) according to Grün [28], and then corrected by the corresponding receiver gain value, number of scans and aliquot mass.

**Table 1:** Acquisition parameters for the X- and Q-band ESR measurements of tooth enamel samples.

Parameter	X-band (9 GHz)	Q-band (34 GHz)
Resonator model	ER 4102ST	ER 5106QT/W
Sample mass (mg)	68 ^_^ 90	2.2 ^_^ 2.4
Inner diameter of sample tube (mm)	4	1
Incident microwave power (mW)	1	2.2
Sweep width (mT)	15	15
HF modulation (kHz)	100	100
Modulation amplitude (mT)	0.1	0.2
Number of points	1024	1320
Time constant (ms)	5.12	10.24
Sweep time (s)	20.48	23.10
Number of scans	1 ^_^ 20	1

The three enamel samples were then measured by Q-band ESR at IRSN with a EMX plus Bruker spectrometer coupled to a cylindrical TE_012_ Q-band cavity (ER5106QT/W). Acquisition parameters are given in **Table 1**. Each aliquot was carefully weighed and a single ESR tube was used (see next section). The mass per aliquot was ranging between ~ 2.20 to ~ 2.40 mg, and a relative variability < 3% was tolerated in the weight of all the aliquots of a given sample. To ensure constant experimental conditions over time, the temperature of the room was kept constant using an air conditioning unit. ESR measurements were performed at room temperature and under a nitrogen atmosphere to avoid variations of humidity over time. For each enamel sample, all the aliquots were measured between 5–10 times (1 scan each) with removal of the tube from the cavity between successive measurements. These conditions resulted in a total measurement time of 4–5 hours for a given sample. This procedure was repeated over 3 different days in order to evaluate the repeatability of the ESR measurements over time. Intensities were extracted from peak-to-peak amplitudes of the ESR signal of enamel (T1-B2), and then corrected by the corresponding number of scans, aliquot mass and Quality (Q)-factor of the cavity. Because the Q-factor could vary from one measurement to another in Q-band, and significantly impact the ESR signal intensities, specific corrections are thus required in order to achieve accurate quantitative results [29].

D_E_ values were calculated by fitting procedures carried out with the Microcal OriginPro 8.5 software using a Levenberg-Marquardt algorithm by chi-square minimisation. Further information may be found in the *Origin 8 User Guide (2007)*. A single saturating exponential (SSE) function was used to fit the experimental data points derived from both X- and Q-band measurements, and data were weighted by the inverse of the squared ESR intensity (see [30] for further details). Fitted parameter errors were assessed through the square root of the covariance matrix diagonal values.

### Optimisation of Q-Band Measurements: Preliminary Tests

Due to the small dimensions of the Q-band resonator and its high sensitivity to any change in the experimental conditions, some preliminary tests were performed with the most irradiated aliquot (D = 5000 Gy) of sample #1 to optimise and standardise the analytical procedure for the dose evaluations.

### Uncertainty associated to differences in glassware

Five quartz tubes (open on one end) with an internal diameter (ID) of 1 mm were selected and the same subsample of the aliquot, corresponding to a mass of ~ 2.20 mg, was successively put into these different tubes (with a relative variability in sample mass among tubes of 1.7%). Each tube was measured 8 times, the tube being removed from the cavity between each repeated measurements. The same procedure was also repeated using 3 different capillary tubes (ID = 0.8 mm) that were sealed on one end by paraffin. In this case, sample mass varied between 2.02 and 2.08 mg from one tube to another (relative variability of 1.6%). All measurements were carried out within 2–3 hours to ensure constant experimental conditions over time.

**Table 2** shows that the variability of the ESR intensities among repeated measurements with each tube gives coefficients of variation (CV) ranging from 0.65 to 4.57%. For the capillary tubes, this variability is between 1.36 and 4.62%, i.e. apparently somewhat slightly higher on average than that obtained with the tubes. Considering the variations from tube to tube, the variability among the mean intensities achieved with the 5 different tubes corresponds to a value of 2.50%, while this is of 2.85% for the capillary tubes. For comparison, a similar study was conducted at X-band using the same sample: 5 different quartz tubes with ID = 3 mm (subsample mass ~ 79 mg) yielded a relative variation of the ESR intensities of 1.34%. When considering each tube, the ESR intensities over repeated measurements gave CV values between 0.42 and 1.22%. These values are significantly lower than those obtained with Q-band measurements, thus indicating a better inherent precision of the ESR intensities afforded by X-band spectroscopy.

**Table 2:** Variability of the ESR intensities performed in Q-band using different quartz tubes and capillary tubes. T1-B2 intensity values were corrected by the corresponding sample mass.

		Tube			Capillary tube		
Tube or Capillary	Number of measurements	ESR intensity (a.u.)	Standard deviation	CV (%)^a^	ESR intensity (a.u.)	Standard deviation	CV (%)^a^
1	8	7.817	0.051	0.65	3.758	0.174	4.62
2	8	7.532	0.205	2.73	3.829	0.110	2.87
3	8	7.530	0.344	4.57	3.973	0.054	1.36
4	8	7.871	0.143	1.82			
5	8	7.446	0.164	2.20			
	**Mean**	7.639		2.39	3.853		2.95
	**Standard deviation**	0.191			0.110		
	**CV (%)**^**a**^	2.50			2.85		

^a^ CV: coefficient of variation (i.e. standard deviation relative to the mean).

To sum up, these results indicate that the influence associated with differences in glassware can hardly be differentiated from the variability of the ESR intensities among repeated measurements, especially when working with a Q-band cavity. This variability is most likely from uncertainties associated with tube positioning within the cavity and preferential orientations of the grains in respect to the magnetic field.

Given these results, we decided to carry out the subsequent Q-band ESR measurements by using tubes instead of capillary tubes; not only because the precision achieved is slightly better, but also for practical reasons. Indeed, the need to manually close one end of the capillary tubes makes the procedure longer and at the same time may limit the precision of the measurements. Furthermore, we decided to run all the Q-band ESR measurements of our study with a single tube to remove the uncertainty that may arise from the heterogeneity in glasswares.

### Effect of sample mass on Q-band cavity response

Sample mass is a crucial parameter to consider for the optimisation of the cavity response, since it may easily be adjusted to improve the signal-to-noise ratio (S/N) for a given sample. Because the relation between sample mass and ESR intensity is known to be not fully linear [6], we performed some tests by varying sample mass in order to evaluate the Q-band cavity response.

**Fig 1:**
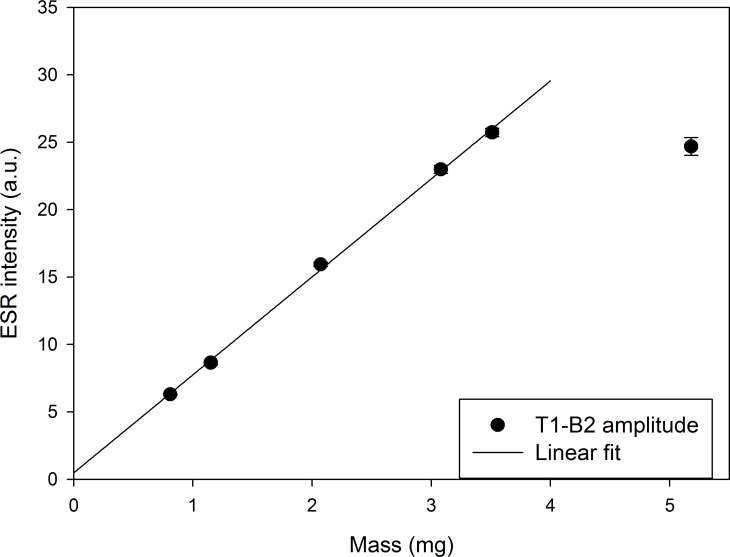
ESR intensity versus sample mass for sample #1 (D = 5 kGy). Experimental uncertainties correspond to one standard deviation from the repeated measurements. Linear regression: y = 0.4838 + 7.2681x. r^2^ = 0.999.

To do so, we selected the most irradiated aliquot of sample #1 and carried out 5 to 8 repeated ESR measurements at increasing mass from 0.8 to 5 mg (corresponding to 1.0 to 5.5 mm of sample height in the tube), with removal of the tube from the cavity between successive measurements. **Fig 1** shows an apparent linear correlation between mass of sample and ESR intensity up to 3.5 mg (sample height of 4 mm when using a tube of ID = 1 mm). For a mass > 3.5 mg (i.e. height > 4 mm) the ESR intensity does not increase anymore, which indicates that the sensitivity of the cavity decreases. This trend is similar to that obtained for X-band cavities [31,32], except that the mass values considered are considerably lower with Q-band, showing thus the higher sensitivity of the resonator. With this test we basically have checked that the maximization of the ESR signal is achieved when the active measuring region of the cavity is filled with sample.

Given these observations, < 3 mg of sample was systematically used for the rest of the study, not only to make sure that the whole sample was fully positioned inside the active region of the Q-band resonator, but also to guarantee a linear relation between sample mass and ESR intensity that allows simple corrections. Nevertheless, as an additional preventive measure, the variation of sample mass from one aliquot to another of a given sample was also carefully limited, so that the impact of this mass correction could be minimised.

## Results and Discussion

### Spectral resolution and sensitivity of X- and Q-band spectrometers

**Fig 2:**
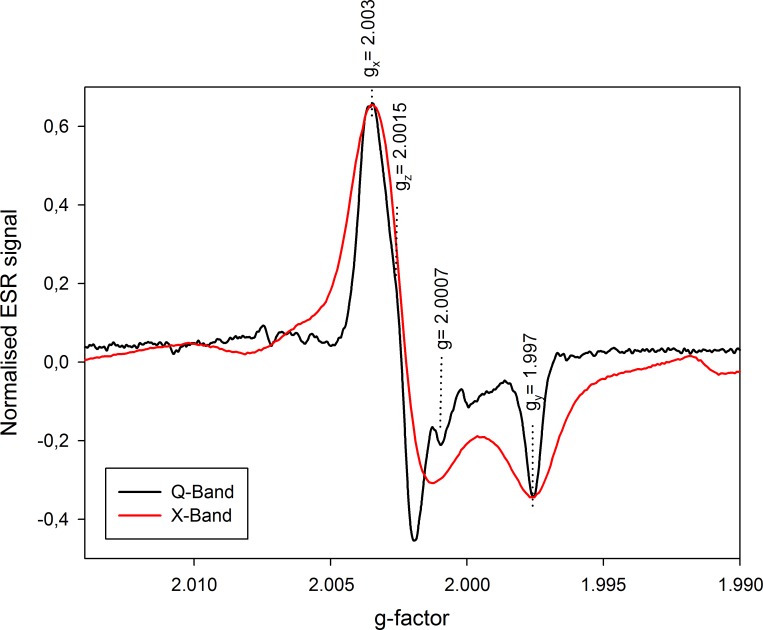
X- and Q-band spectra of the powder tooth enamel sample #3 (natural aliquot). Spectra are recorded with the same acquisition parameters: modulation frequency = 100 KHz; modulation amplitude = 2 G; microwave power = 2 mW. ESR intensities were normalised according to peak-to-peak T1-B2 amplitude.

**Fig 2** shows the hydroxyapatite ESR spectra obtained for powder fossil tooth enamel of sample #3 (natural aliquot) as recorded by both X- and Q-band cavities with the same acquisition parameters. It should be noted that the sensitivity in Q-band ESR is considerably increased in comparison with that achieved in X-band. In general, sensitivity is defined as the ability to detect small changes in the quantity that is being measured (in ESR: number of electron spins that can be detected; evaluated via the measurement of the signal amplitude). In that regard, the signal-to-noise ratio could be used as a reliable proxy to estimate the sensitivity of a cavity. A S/N ratio of 200 using 80 mg of powder tooth enamel was obtained from the X-band spectrum shown in **Fig 2**, whereas the Q-band spectrometer yielded a S/N of 110 with only 2.2 mg of sample. This means that Q-band measurements are ~ 20 times more sensitive than similar X-band measurements. This is actually consistent with other previous studies that have shown a gain in sensitivity by a factor of 50 for enamel biopsy samples [13].

However, the most striking observation is perhaps the significantly higher spectral resolution of the different components of the hydroxyapatite signal achieved with Q-band in comparison with the X-band resonator (**Fig 2**). For example, the signal at g ~ 2.0007, usually attributed to an isotropic CO_2_ˉ contribution [9,10], is hidden in the main composite signal of the X-band spectrum whereas it can be identified and resolved using Q-band.

**Fig 3:**
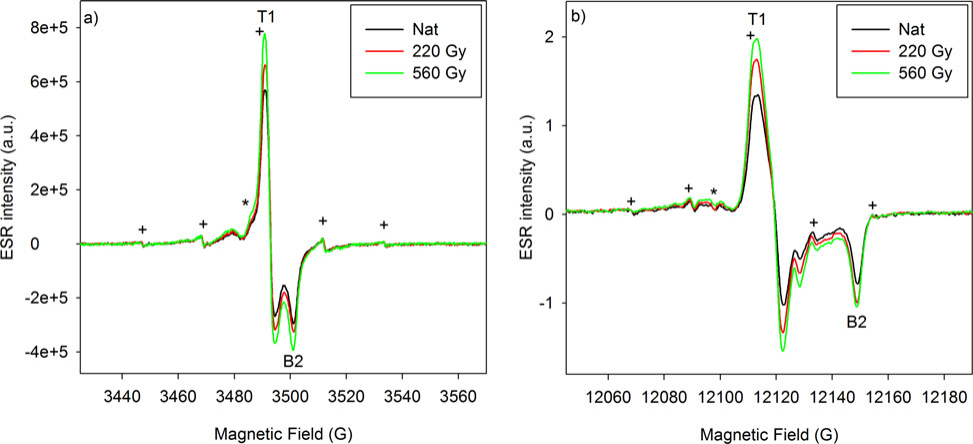
**X-band (a) and Q-band (b) spectra of the powder sample #3 (natural aliquot + two irradiated aliquots).** Acquisition parameters are provided in **Table 1**. Sweep width is of 145 G for all ESR spectra. Key: (*) identifies the SO_2_ˉ component. The multiplet centered at g = 2.0033 (labelled as “+”) is an isotropic and non-radiosensitive signal due to a dimethyl radical [9,21], and the signal at g = 2.0115 is probably associated to a CO_3_ˉ radical [36].

**Fig 3** shows another example of X- and Q-band spectra from the Early Pleistocene sample #3, highlighting again the difference in signal resolution: T1 and B2 are only separated by 10 G in X-band spectroscopy vs. almost 36 G in Q-band. In addition, it could be observed that the signal at g = 2.0056 (labelled *), which is probably attributed to an isotropic SO_2_ˉ component [3,33], can be isolated in the Q-band spectra, contrary to the X-band ones. Consequently, in the light of the recent results obtained on tooth enamel fragments [34,35], this higher resolution might be especially useful for the identification and differentiation of the different components of the main composite signal, in particular the orientated CO_2_ˉ and the non-orientated CO_2_ˉ radicals at the origin of the ESR signal of hydroxyapatite. This aspect will need to be further explored in the future.

### Variability of the ESR intensities over repeated measurements of the DRCs

The main objective of the next sections is to gain knowledge about the precision of the ESR measurements using a Q-band spectrometer and to evaluate its impact on the resulting D_E_ values. Three different samples that cover a wide range of chronologies were selected to perform this study.

#### X-band measurements

All the aliquots of a given sample (10–14 aliquots) were successively measured according to the experimental conditions described in **materials and methods**. This procedure was repeated over four different days to evaluate the variability of the results. Consequently, a mean ESR intensity value, a standard deviation and a CV were obtained for each aliquot. Then, a mean CV value was calculated by averaging the CV values from each aliquot of a given sample. These values are between 0.96% for sample #3 and 1.25% for sample #1, i.e. fairly consistent with the results from previous studies [5,37].

#### Q-band measurements

Each aliquot of a given sample (10–14 aliquots) was measured between 5–10 times (1 scan each). The tube was systematically removed from the cavity between each successive measurement. This operation was repeated over three different days. The variability of the resulting ESR intensities of a given aliquot over successive measurements may be used as a proxy to evaluate the repeatabilility of the Q-band measurements (see summary in **Table 3**): CV values range from a minimum value of 0.65% (aliquot 10, day 1) to a maximum one of 5.96% (aliquot 4, day 2) for sample #1, from 0.55 to 3.54% for sample #2 and from 0.54 to 7.24% for #3, with mean values shown in column 5 of **Table 3**. These results confirm that the inherent precision of Q-band measurements is lower than that of X-band. Additionally, this quite high variability indicates that several measurements are required to achieve a meaningful intensity value for each aliquot.

**Table 3:** Variability of the ESR intensities derived from repeated Q-band measurements performed on each aliquot of a given sample.

Sample	day	Number of aliquots	Number of replicate measurements	ESR intensity: mean CV (%)	ESR intensity: min. CV (%)	ESR intensity: max. CV (%)
#1	day 1	10	5 ^_^ 7	3.07	0.65	5.89
	day 2	10	9 ^_^ 10	3.20	1.78	5.96
	day 3	10	9	1.93	0.78	5.05
#2	day 1	10	9	1.30	0.56	3.54
	day 2	10	9	1.23	0.55	2.52
	day 3	10	9	2.40	0.90	4.22
#3	day 1	14	9 ^_^ 10	2.56	0.78	7.24
	day 2	14	9	1.75	0.82	3.13
	day 3	14	9	2.25	0.54	4.24

For each aliquot of a given sample, repeated ESR measurements lead to the calculation of a mean ESR intensity with an associated coefficient of variation (CV). The mean CV indicated in column 5 is obtained by averaging the CV achieved for each aliquot of a given sample within a specific day. Maximum and minimum CV values observed for each sample within a specific day are indicated in column 6 and 7, respectively.

To summarise, although a strict comparison is difficult because of slightly different analytical procedures, our results nevertheless suggest that the precision achieved with the Q-band resonator is significantly lower than that afforded by X-band ESR. This difference could be mainly explained by the high sensitivity of the Q-band resonator, which makes the signal more vulnerable to small changes in experimental conditions, such as sample positioning in the resonator and redistribution of the enamel powder in the sample tube. In addition, the high sensitivity of this resonator makes crucial to keep stable the laboratory environmental conditions (i.e. temperature and humidity) to ensure a good stability of the spectrometer over time.

### Comparison of the DRCs

**Fig 4:**
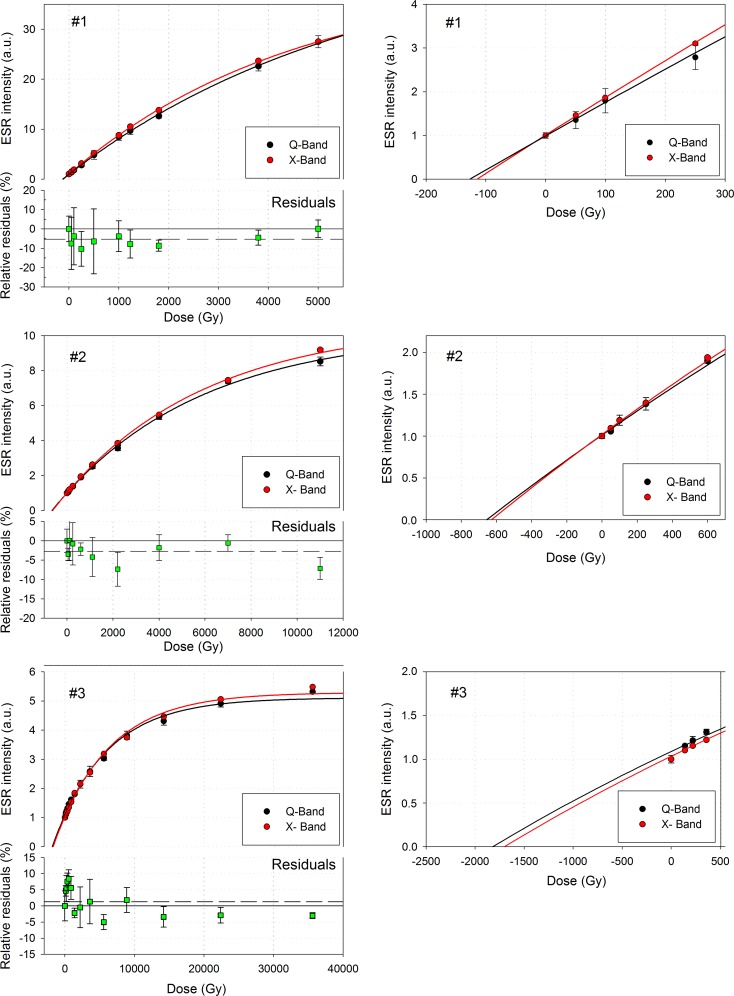
Comparison of the X- and Q-band DRCs. ESR intensities were normalised according to the natural point of each DRC. Relative residual (%) are defined as the relative difference between the two points for a given dose. X-band measurements are taken as references. A zoom for low doses of each DRC is shown on the right part of the figure.

Final ESR intensities were calculated by averaging the mean intensity values of each aliquot derived from the different days of measurements. Final DRCs from X- and Q-band measurements are shown in **Fig 4**.

To enable comparisons, intensities were systematically normalised to that of the corresponding natural aliquot, which is supposed to be stable over several millions of years [7] and may thus be considered as a reliable ESR intensity standard. It could be observed that Q-band ESR intensities for samples #1 and #2 are on average lower than those measured by X-band spectroscopy, by between -5.29 ± 3.52% (#1) and -2.72 ± 2.75% (#2), while they are higher for sample #3 (1.26 ± 4.40%). In addition, it seems that Q-band measurements produce slightly lower intensities than X-band when only focusing on doses > 1.5 kGy, with a drop in the intensities values of -1.67 ± 2.60% (#3), -4.19 ± 3.53% (#2) and -4.40 ± 4.38% (#1). However, the magnitude of the associated errors illustrates a large variability of the values from one aliquot to another of a given sample (**Fig 4**). Consequently, this associated error makes difficult to draw any definitive conclusion and this systematic trend may very likely not be significant. Finally, because X-band analyses have been performed 13 months (sample #1) and 32 months (samples #2 and #3) before the Q-band measurements, the hypothesis of a slight anomalous fading of the signal over time might be envisaged, but previous studies do no have provided conclusive results on this question [5,38]. In addition, ESR measurements of the same samples performed with the X-band spectrometer at CENIEH did not show any significant decrease in the signal intensity over 1–2 years.

### Impact on the D_E_ values

For each sample, several DRCs, 4 for X-band and 3 for Q-band ESR spectroscopy, were derived from the repeated measurements performed on different days. A SSE function was fitted through each DRC of the different samples and the data were weighed by the inverse of the squared ESR intensity (1/I^2^), giving the values shown in **Table 4.**

**Table 4:** Fitting results obtained from X- and Q-band measurements of the three samples. A SSE function with data weighting by 1/I^2^ was used.

Sample		#1			#2			#3		
		D_E_ (Gy)	±	Adjusted r^2^	D_E_ (Gy)	±	Adjusted r^2^	D_E_ (Gy)	±	Adjusted r^2^
X-Band	day 1	113.7	1.8	0.9998	623	21	0.9987	1713	101	0.9945
	day 2	116.5	3.0	0.9993	653	25	0.9983	1676	91	0.9953
	day 3	113.7	3.3	0.9992	627	23	0.9985	1695	106	0.9938
	day 4	113.5	1.3	0.9999	612	18	0.9990	1715	93	0.9953
	Final D_E_^a^	114.4	1.5	0.9998	628	18	0.9991	1700	94	0.9952
Q-Band	day 1	128	15	0.9871	636	55	0.9911	1989	158	0.9895
	day 2	116	12	0.9901	707	29	0.9980	1741	187	0.9825
	day 3	137	12	0.9929	629	53	0.9942	1747	141	0.9896
	Final D_E_^a^	126	5	0.9984	655	30	0.9975	1820	139	0.9907

^a^ Final D_E_ corresponds to the fitting results derived from the mean ESR intensities considering the different days.

The results indicate that the reproducibility of the D_E_ values is systematically better with X-band spectroscopy compared to Q-band. For sample #1, D_E_s range from 113.5 ± 1.3 to 116.5 ± 3.0 Gy with the X-band resonator, against D_E_ values between 116 ± 12 and 137 ± 12 Gy using Q-band (**Table 4**). This variability is also high for sample #3, where the D_E_ values derived from X-band are all around 1700 Gy, while they are between 1741 ± 187 and 1989 ± 158 Gy in Q-band. Actually, the relative variability in the D_E_s over repeated measurements is ranging from 1.1% (sample #3) to 2.8% (sample #2) in X-band, while this is from 6.6% (sample #2) to 8.2% (sample #1) in Q-band. The higher scatter in the D_E_ values achieved with the Q-band spectrometer is mainly explained by the larger experimental uncertainty in the ESR intensities (**Table 3**) in comparison with the X-band measurements. However, despite this variability, it is worth noting that the D_E_s derived from Q-band measurements performed on different days for a given sample are all consistent at 1 sigma (**Table 4**). The same observation is done for X-band measurements.

Final D_E_ values were derived from the SSE function fitted through the final DRC of each sample (i.e. mean ESR intensities of each aliquot derived from the different days of measurements). The comparison of X- and Q-band final D_E_s shows that the results are consistent at 1 sigma for samples #2 and #3, and at 2 sigma for sample #1 (**Table 4**), suggesting that both ESR spectroscopies could thus independently be used for dose estimation of fossil tooth enamel.

However, one may also observe that Q-band measurements give slightly higher final D_E_ values than with the X-band resonator (**Table 4** and zoom on low doses in **Fig 4**), about 10.1, 4.3 and 7.0% for samples #1, #2 and #3, respectively. Nevertheless, the limited number of samples in the present study (n = 3) prevent from determining whether this apparent systematic trend is real or not. Further work is definitely needed in the future to address this question.

Finally, calculated adjusted r^2^ values could provide an indication of the goodness-of-fit afforded by the SSE function through the experimental data points. Although results show overall good fittings for all the samples (adjusted r^2^ values > 0.98), it is nevertheless worth mentioning that adjusted r^2^ values are slightly better for X-band measurements (**Table 4**). The lowest values are overall obtained for sample #3, which is very likely explained by the shape of the DRC and the relative difficulty for the SSE function to fit the dataset from Early Pleistocene samples [5,30].

The magnitude of the D_E_ error may also be used to evaluate the reliability and precision of the fitting. In that regard, we observed that the relative errors in the D_E_ values are higher for the oldest sample (#3), which is in agreement with previous comments. When comparing the D_E_ errors derived from X and Q-band measurements, the first ones yield systematically lower errors than the latter (up to a factor of two), which is consistent with the calculated adjusted r^2^ values (**Table 4**). For example, sample #1 gives a relative error of 1.3% in X-band versus 4.0% in Q-band; and sample #3 provides error values of 5.5 and 7.6% for X- and Q-band, respectively.

Finally, in order to minimise any source of uncertainties that may arise from different laboratory conditions for X- and Q-band measurements, samples #2 and #3 were also analysed at IRSN with the same spectrometer and protocol used for Q-band measurements but using a X-band cavity (ER SHQE4122) and ~ 60 mg per aliquot. From these new measurements, we obtained a D_E_ value of 654 ± 18 Gy (r^2^ = 0.9992) for sample #2 and 1627 ± 70 Gy (r^2^ = 0.9971) for sample #3. These results are consistent with the X-band measurements carried out at the CENIEH (**Table 4**). They suggest that the spectrometer and laboratory conditions are not at the origin of the slight differences observed between Q-band and X-band results.

## Conclusions

This work provides one of the first overviews of the potential and limitations of Q-band ESR spectroscopy for dose reconstruction of fossil tooth enamel.

Our results show that Q-band offers a much higher sensitivity (~ 20 times) in comparison with X-band: a sample mass of only a few milligrams is enough to achieve a good S/N. In addition, the resolution of the spectral components is significantly improved, which may be especially useful for developing more accurate dose assessments of the composite radiation induced ESR signal of fossil enamel, in particular for highly valuable fossil remains. Other Quaternary materials with composite ESR signals such as corals, speleothems or molluscs may also beneficiate from this increased resolution and sensitivity offer by Q-band resonators.

On the other hand, Q-band ESR dose evaluation has several limitations. Our results overall indicate a lower measurement precision and thus a higher D_E_ scatter over repeated measurements in comparison with X-band. Nevertheless, all the D_E_ values derived from X- and Q-band measurements are within error. These results demonstrate thus that Q-band may also be used for the dose reconstruction of fossil tooth enamel samples covering the whole Pleistocene.

Finally, although this Q-band study is limited to a reduced number of samples, the results achieved are promising and demonstrate the potential of Q-band spectroscopy in the field of dating/dosimetry of fossil tooth enamel, especially when working with valuable tooth samples. However, for the moment, X-band remains preferred for routine dose determinations because of the more precise measurements combined with a lower equipment cost.

## References

[1] Ikeya M. Dating a stalactite by electron paramagnetic resonance. Nature. 1975; 255: 48–50.

[2] Grün R. Electron Spin Resonance (ESR) dating. Quat Int. 1989; 1: 65–109.

[3] Ikeya M, Zimmerman M, Whitehead N. New applications of electron spin resonance-dating, dosimetry and microscopy Singapore: World Scientific Publishing Co Pte Ltd 1993.

[4] Rink WJ. Electron spin resonance (ESR) dating and ESR applications in Quaternary science and archaeometry. Radiat Meas. 1997; 27: 975–1025.

[5] Duval M, Guilarte Moreno V, Grün R. ESR Dosimetry of fossil enamel: some comments about measurements precision, long-term signal fading and dose-response fitting. Radiat Prot Dosim. 2013; 157(4): 463–476. 10.1093/rpd/nct167 23832975

[6] Fattibene P, Callens F. EPR dosimetry with enamel: A review. Appl Radiat Isot. 2010; 68: 2033–2116. 10.1016/j.apradiso.2010.05.016 20599388

[7] Schwarcz HP. ESR study of tooth enamel. Nucl Tracks. 1985; 10: 865–867.

[8] Misra SK. Multifrequency Electron Paramagnetic Resonance: theory and applications Weinheim, Germany: Wiley-VCH; 2011 p.1056.

[9] Bouchez R, Cox R, Herve A, Lopez-Carranza E, Ma JL, Piboule M, et al Q-Band ESR studies of fossil teeth: consequences for ESR dating. Quat Sci Rev. 1988; 7: 497–501.

[10] Callens F, Vanhaelewyn G, Matthys P. Some recent multi-frecuency electron paramagnetic resonance results on systems relevant for dosimetry and dating. Spectrochim Acta A. 2002; 58: 1321–1328. 10.1016/s1386-1425(01)00721-1 11993479

[11] Vanhaelewyn GCAM, Sadlo J, Matthys PFAE, Callens FJ. Comparative X- and Q-band EPR study of radiation-induced radicals in tooth enamel. Radiat Res. 2002; 158: 615–625. 12385639 10.1667/0033-7587(2002)158[0615:cxaqbe]2.0.co;2

[12] Romanyukha A, Mitchell CA, Schauer DA, Romanyukha L, Swartz HM. Q-band EPR biodosimetry in tooth enamel microsamples: feasiblility test and comparison with X-band. Health Phys. 2007; 93(6): 631–635. 17993843 10.1097/01.HP.0000269507.08343.85

[13] De T, Romanyukha A, Trompier F, Pass B, Prabhakar M. Feasibility of Q-band EPR dosimetry in biopsy samples of dental enamel, dentine and bone. Appl Magn Reson. 2013; 44: 375–387.

[14] Romanyukha A, Trompier F, Reyes RA. Q-band electron paramagnetic resonance dosimetry in tooth enamel: biopsy procedure and determination of dose detection limit. Radiat Environ Biophys. 2014; 53: 305–310. 10.1007/s00411-013-0511-8 24442862

[15] Jonas M, Grün R. Q-band ESR studies of fossil tooth enamel: implications for spectrum deconvolution and dating. Radiat Meas. 1997; 1: 49–58.

[16] Trompier F, Queinnec F, Bey E, De Revel T, Lataillade JJ, Clairand I, et al EPR retrospective dosimetry with fingernails: Report on first application cases. Health Phys. 2014; 106(6): 798–805. 10.1097/HP.0000000000000110 24776914

[17] Trompier F, Romanyukha A, Reyes R, Vezin H, Queinnec F, Gourier D. State of the art in nail dosimetry: free radicals identification and reactions mechanisms. Radiat Environ Biophys. 2014; 53: 291–303. 10.1007/s00411-014-0512-2 24469226 PMC3996284

[18] Romanyukha A, Trompier F, Reyes R A, Melanson MA. EPR measurements of fingernails in Q-band. Radiat Meas. 2011; 46: 888–892.

[19] Skinner AR, Chasteen ND, Shao JL, Goodfriend GA, Blackwell BAB. Q-band ESR spectra as indicators of fossilization in tooth enamel. Quat Int. 2005; 135: 13–20.

[20] Skinner AR, Chasteen ND, Shao J, Blackwell BAB. Q-band studies of the ESR signal in tooth enamel. Quat Sci Rev. 2001; 20: 1027–1030.

[21] Vanhaelewyn G, Callens F, Grün R. EPR spectrum deconvolution and dose assessment of fossil tooth enamel using maximum likelihood common factor analysis. Appl Radiat Isot. 2000; 52: 1317–1326. 10836449 10.1016/s0969-8043(00)00090-7

[22] Skinner AR, Blackwell B AB, Chasteen N D, Shao J, Min S. Improvements in dating tooth enamel by ESR. Appl Radiat Isot. 2000; 52: 1337–1344. 10836451 10.1016/s0969-8043(00)00092-0

[23] Faivre JPh, Maureille B, Bayle P, Crevecoeur I, Duval M, Grün R, et al The Middle Pleistocene site of Tourville-la-Rivière (Normandy, France): Human Settlement and Human Remains from Northwestern Europe. PLoS ONE. 2014; 9(10): e104111 10.1371/journal.pone.0104111 25295956 PMC4189787

[24] Santonja M, Pérez-González A, Domínguez-Rodrigo M, Sesé C, Soto E, Panera J, et al The Middle Paleolithic site of Cuesta de la Bajada (Teruel, Spain): a perspective on the Acheulean and Middle Paleolithic technocomplexes in Europe. J Archaeol Sci. 2014; 49: 556–571.

[25] Duval M, Falguères C, Bahain JJ, Grün R, Shao Q, Aubert M, et al The challenge of dating Early Pleistocene fossil teeth by the combined uranium series-electron spin resonance method: the Venta Micena palaeontological site (Orce, Spain). J Quatern Sci. 2011; 26(6): 603–615.

[26] Grün R, Rhodes EJ. Simulations of saturating exponential ESR/TL dose response curves-weighting of intensity values by inverse variance. Ancient TL. 1992; 10(3): 50–56.

[27] Duval M. Evaluation du potentiel de la méthode de datation par Résonance de Spin Electronique (ESR) appliquée aux gisements du Pléistocène inférieur: étude des gisements d’Orce (bassin de Guadix-Baza, Espagne) et contribution à la connaissance des premiers peuplements de l’Europe. PhD Thesis, Muséum National d'Histoire Naturelle, Paris. 2008.

[28] Grün R. Methods of dose determination using ESR spectra of tooth enamel. Radiat Meas. 2000; 32(5–6): 767–772.

[29] Eaton GR, Eaton SS, Barr DP, Weber RT. Quantitative EPR. Wien-New York: Springer; 2010 p185.

[30] Duval M, Grün R, Falguères C, Bahain JJ, Dolo JM. ESR dating of lower Pleistocene fossil teeth: limits of the single saturating exponential (SSE) function for the equivalent dose determination. Radiat Meas. 2009; 44(5–6): 477–482.

[31] Zhumadilov K, Ivannikov A, Skvortsov V, Stepanenko V, Zhumadilov Z, Endo S, et al Tooth Enamel EPR Dosimetry: Optimization of EPR Spectra Recording Parameters and Effect of Sample Mass on Spectral Sensitivity. J Radiat Res. 2005; 46(4): 435–442. 16394634 10.1269/jrr.46.435

[32] Hayes RB, Haskell EH, Barrus JK, Kenner GH, Romanyukha AA. Accurate EPR radiosensitivity calibration using small sample masses. Nucl Instrum Meth A. 2000; 441: 535–550.

[33] Barabas M. The nature of the paramagnetic centres at g = 2.0057 and g = 2.0031 in marine carbonates. Nucl Tracks Radiat Meas. 1992; 20: 453–464.

[34] Grün R, Joannes-Boyau R, Stringer C. Two types of CO_2_ˉ radicals threaten the fundamentals of ESR dating of tooth enamel. Quat Geochronol. 2008; 3: 150–172.

[35] Joannes-Boyau R, Grün R. A comprehensive model for CO_2_ˉ radicals in fossil tooth enamel: Implications for ESR dating. Quat Geochronol. 2011; 6: 82–97.

[36] Callens FJ, Verbeeck RMH, Naessens DE, Matthys PFA, Boesman ER. Effect of carbonate content on the ESR spectrum near g = 2 of carbonated calciumapatites synthesized from aqueous media. Calcif Tissue Int. 1989; 44: 114–124. 2537133 10.1007/BF02556470

[37] Duval M. Evaluating the accuracy of ESR dose assessment of pseudo-Early Pleistocene fossil tooth enamel with dose revovery tests. Radiat Meas. 2015; 79: 24–32.

[38] Grün R, Ward K. A long-term fading study for ESR intensity measurement and dose evaluation on fossil tooth enamel. Radiat Meas. 2002; 35(3): 269–274.

